# Probiotic Potential and Safety Assessment of Type Strains of *Weissella* and *Periweissella* Species

**DOI:** 10.1128/spectrum.03047-22

**Published:** 2023-02-27

**Authors:** Francesca Fanelli, Marco Montemurro, Michela Verni, Antonella Garbetta, Anna Rita Bavaro, Daniele Chieffi, Gyu-Sung Cho, Charles M. A. P. Franz, Carlo Giuseppe Rizzello, Vincenzina Fusco

**Affiliations:** a National Research Council, Institute of Sciences of Food Production (CNR-ISPA), Bari, Italy; b Department of Soil, Plant and Food Science, University of Bari Aldo Moro, Bari, Italy; c Max Rubner-Institut, Department of Microbiology and Biotechnology, Kiel, Germany; d Department of Environmental Biology, Sapienza University of Rome, Rome, Italy; University of Mississippi

**Keywords:** adhesion, *Periweissella*, *Periweissella beninensis*, *Weissella*, antibiotic resistance, autoaggregation, cell adhesion, genomics, hemolysis, hydrophobicity, probiotic, safety assessment

## Abstract

Although numerous strains belonging to the *Weissella* genus have been described in the last decades for their probiotic and biotechnological potential, others are known to be opportunistic pathogens of humans and animals. Here, we investigated the probiotic potential of two *Weissella* and four *Periweissella* type strains belonging to the species Weissella diestrammenae, Weissella uvarum, Periweissella beninensis, Periweissella fabalis, Periweissella fabaria, and Periweissella ghanensis by genomic and phenotypic analyses, and performed a safety assessment of these strains. Based on the results of the survival to simulated gastrointestinal transit, autoaggregation and hydrophobicity characteristics, as well as adhesion to Caco-2 cells, we showed that the *P. beninensis*, *P. fabalis*, *P. fabaria*, *P. ghanensis*, and *W. uvarum* type strains exhibited a high probiotic potential. The safety assessment, based on the genomic analysis, performed by searching for virulence and antibiotic resistance genes, as well as on the phenotypic evaluation, by testing hemolytic activity and antibiotic susceptibility, allowed us to identify the *P. beninensis* type strain as a safe potential probiotic microorganism.

**IMPORTANCE** A comprehensive analysis of safety and functional features of six *Weissella* and *Periweissella* type strains was performed. Our data demonstrated the probiotic potential of these species, indicating the *P. beninensis* type strain as the best candidate based on its potential probiotic features and the safety assessment. The presence of different antimicrobial resistance profiles in the analyzed strains highlighted the need to establish cutoff values to perform a standardized safety evaluation of these species, which, in our opinion, should be mandatory on a strain-specific basis.

## INTRODUCTION

The genus *Weissella* comprises a group of lactic acid bacteria (LAB), which have received increased attention in the last decade mainly for their biotechnological and probiotic potential (1–3). The taxonomy of this genus, which belongs to the phylum *Firmicutes*, order *Lactobacillales*, and the family *Lactobacillaceae*, was recently updated by Fanelli et al. (2), who, based on a comprehensive phylogenomic analysis, clustered the 21 *Weissella* species described to date into six species groups. Furthermore, Bello et al. (4) recently revised the taxonomy of the family *Leuconostocaceae* merged with the *Lactobacillaceae* (5) by phylogenomic and comparative genomic analysis. By using a 16S rRNA phylogenetic and a phylogenomic tree based on the concatenated sequences of 498 core proteins from the genera *Convivina*, *Fructobacillus*, *Leuconostoc*, *Oenococcus*, and *Weissella*, the authors amended the description of the *Weissella* genus by excluding 5 species, which were reclassified in the proposed novel genus *Periweissella*: P. cryptocerci (basonym W. cryptocerci [6]), P. beninensis (basonym W. beninensis [7]), P. fabalis (basonym W. fabalis [8]), P. fabaria (basonym W. fabaria [9]), and P. ghanensis (basonym W. ghanensis [10]).

Despite their widespread occurrence in several food matrices and fermentative processes, the role of *Weissella* species is controversial. Indeed, the recent association of weissellas with human infections, although rare, in individuals with reduced immunity has raised concern related to the use of these species in food industries (1, 11). In aquaculture, Weissella ceti has recently been recognized as the etiological agent of the so-called “weissellosis” (12, 13), a disease occurring in farmed rainbow trout causing septicemia with a high mortality rate (14).

The pathogenic potential of this genus was also inferred by the analysis of genomic sequences, which identified a limited number of virulence determinants, such as hemolysin genes (11, 15). These genes, however, are ubiquitously present in many LAB, and their role in pathogenicity is still unknown. A further concern relating to their safety is the occurrence of antibiotic resistance in several *Weissella* strains. Numerous studies reported resistance of food-associated *Weissella* spp. to antibiotics such as vancomycin, gentamicin, streptomycin, and norfloxacin (16–18). Thus, a strain-specific safety assessment should be mandatory for the use of *Weissella* strains in food industries or as probiotics.

To date, none of the *Weissella* strains are recognized as GRAS (generally recognized as safe) by the Food and Drug Administration (FDA) in the United States, nor have been included in the QPS (qualified presumption of safety) list by the European Food Safety Authority (EFSA). In order to improve the knowledge related to the exploitation of the biotechnological potential of *Weissella* and *Periweissella* species, we investigated the probiotic potential of six recently sequenced *Weissella* and *Periweissella* type strains by evaluating exopolysaccharide (EPS) production, survival under gastrointestinal conditions, adhesion, autoaggregation capacity, cell surface hydrophobicity, and antimicrobial activity of each strain. We also identified and predicted functional properties of homologs of probiotic genes described in lactic acid bacteria (19). Moreover, we performed an *in vitro* and *in silico* safety assessment by using whole-genome sequencing (WGS) data to infer the risk assessment of these potential probiotics, as recently discussed by Peng et al. (20).

## RESULTS

### *In silico* analysis.

**(i) Genes related to probiotic and nutritional function.** Genes encoding potential probiotic and nutritional functions identified in the *Weissella* and *Periweissella* type strains used in this study are shown in Table 1. All strains harbored the housekeeping gene *groEL* (with the predicted protein having an average identity with the RefSeq CCC15170.1 of 74%) and the *LBA1446* gene (with the predicted protein having an average identity with the RefSeq AAV43270.1 of 50%) coding for a major facilitator superfamily (MFS) transporter. The linoleate isomerase gene was present in all the strains (with the predicted protein having an average identity with the RefSeq CBY45494.1 of 43%), with the exception of Weissella uvarum B18NM42^T^. The d-alanine transfer protein gene and the *LBA1679* gene coding for an ATP-binding cassette (ABC) transporter were found in all (with the predicted protein having an average identity with the RefSeq CBY45494.1 of 35%) but not in *P. beninensis* LMG 25373^T^. Homologs of the oligo-1,6-glucosidase and ɑ-glucosidase encoded by *malL* and *agl* genes were identified and had the same hit in *P. fabalis* LMG 26217^T^, *P. fabaria* LMG 24289^T^, *P. ghanensis* DSM 19935^T^, and *W. uvarum* B18NM42^T^. The *folP* and *folK* genes were absent in *P. beninensis* LMG 25373^T^ and Weissella diestrammenae DSM 27940^T^, but a complete riboflavin biosynthetic cluster was identified in *P. fabalis* LMG 26217^T^, *P. fabaria* LMG 24289^T^, and *P. ghanensis* DSM 19935^T^. Homologs of the *LBA1432* gene, coding for a GTP pyrophosphokinase family protein, were annotated (with the predicted protein having an average identity with the RefSeq AAV43257.1 of 35%) in *P. fabalis* LMG 26217^T^, *P. ghanensis* DSM 19935^T^, and *W. uvarum* B18NM42^T^.

**TABLE 1:** Genes related to probiotic and nutritional functions in *Periweissella* and *Weissella* strains^*a*^

General function	Product	Gene	*P. beninensis* LMG 25373^T^	*W. diestrammenae* DSM 27940^T^	*P. fabalis* LMG 26217^T^	*P. fabaria* LMG 24289^T^	*P. ghanensis* DSM 19935^T^	*W. uvarum* B18NM42^T^
CLA synthesis	Linoleate isomerase	*pai*	KAK10_07600	KAR27_05835	KAR41_09210	KAR50_09555	KAR53_00640	NA

pH survival	Heat shock protein 60	*groEL*	KAK10_03115	KAR27_07485	KAR41_03335	KAR50_07615	KAR53_09630	KAR63_02380
	d-Alanine transfer protein	*dltD*	NA	KAR27_05275	KAR41_04845	KAR50_01215	KAR53_04680	KAR63_01905
	Amino acid antiporter	*La*57	NA	KAR27_03615	KAR41_09275	NA	KAR53_05910	KAR63_05510

Bile salt survival	MFS transporter	*LBA1446*	KAK10_07025	KAR27_07200	KAR41_07660	KAR50_07235	KAR53_01010	KAR63_04340
	ABC transporter	*LBA1679*	NA	KAR27_07955	KAR41_06620	KAR50_06075	KAR53_02330	KAR63_06120
	GTP pyrophosphokinase family protein	*LBA1432*	NA	NA	KAR41_02285	NA	KAR53_03555	KAR63_04950

Synthesis of vitamins	Dihydropteroate synthase/dihydropteroate pyrophosphorylase	*folP*	NA	NA	KAR41_07615	KAR50_07285	KAR53_10090	KAR63_02165
	2-Amino-4-hydroxy-6-hydroxymethyldihydropteridine diphosphokinase	*folK*	NA	NA	KAR41_07600	KAR50_07300	KAR53_10105	KAR63_02150
	Riboflavin biosynthetic cluster	*ribABCDEFHU*	NA	NA	KAR41_01765-01780, KAR41_04250, KAR41_06605	KAR50_01690-01705, KAR50_01585, KAR50_06090	KAR53_07575-07590, KAR53_04320, KAR53_02345	NA

Starch metabolism	Oligo-1,6-glucosidase/ɑ-glucosidase	*malL*/*agl*	NA	NA	KAR41_06265	KAR50_01875	KAR53_01435	KAR63_01965

a ^T^, type strain; NA, not annotated; CLA, conjugated linoleic acid.

**(ii) Virulence determinants.** Virulence determinants present in the genomic sequences of *Weissella* and *Periweissella* type strains are reported in Table 2. According to the *in silico* analysis, each genome harbors hemolysin genes, including a hemolysin III family protein gene, the HlyC/CorC family transporter, and the methyltransferase TlyA gene. The TlyA protein has a controversial function as a virulence factor in Mycobacterium tuberculosis, and its role has been recently questioned by Arenas et al. (21). The *in silico* analysis performed by these researchers suggested that TlyA is involved in ribosomal biogenesis and that there is a functional annotation error regarding this protein family in several microbial and plant genomes. In addition, the *cvb* gene coding for the conserved virulence factor B, a DNA-binding protein, which in Staphylococcus aureus contributes to the expression of virulence factors and to pathogenicity and is involved in the production of hemolysin, DNase, and proteases (22), was identified in each of the analyzed genomes. Similarly, the gene coding for the fibronectin-binding protein PavA, essential for virulence in Streptococcus pneumoniae (23), was also identified as occurring in each genome.

**TABLE 2:** Virulence determinants in *Periweissella* and *Weissella* strains (see “Bioinformatic methods” for details)^*a*^

Virulence mechanism	Gene/product	*P. beninensis* LMG 25373^T^	*W. diestrammenae* DSM 27940^T^	*P. fabalis* LMG 26217^T^	*P. fabaria* LMG 24289^T^	*P. ghanensis* DSM 19935^T^	*W. uvarum* B18NM42^T^
Adhesion	*pavA*	KAK10_02630	KAR27_01820	KAR41_06460	KAR50_06270	KAR53_02520	KAR63_05020

Hemolysis	HlyC/CorC family transporter	KAK10_08715	KAR27_00030	KAR41_07960	KAR50_06905	KAR53_09880	KAR63_02740
	Hemolysin III family protein	KAK10_02545	KAR27_07725	KAR41_06570	KAR50_06125	KAR53_02380	NA
	Hemolysin A/rRNA methyltransferase TlyA	KAK10_05850	KAR27_07810	KAR41_03085	KAR50_07935	KAR53_04010	KAR63_02500
	HlyC/CorC family transporter	KAK10_01065	NA	KAR41_02635	KAR50_04885	KAR53_01620	KAR63_02745

Regulation of virulence factors	*cvb*	KAK10_02540	KAR27_01505	KAR41_06575	KAR50_06120	KAR53_02375	KAR63_04120
	*arl*S	KAK10_08770	KAR27_04665	KAR41_02765/KAR41_08025	KAK50_06830/KAR50_08245	KAR53_07545/KAR53_09950	KAR63_05500
	*arl*R	KAK10_08775	KAR27_04660	KAR41_02770/KAR41_08030	KAR50_06835/KAR50_08240	KAR53_07550/KAR53_09955	KAR63_05495
	SaeR response regulator transcription factor	NA	NA	KAR41_07110	KAR50_05355	KAR53_03080	NA
	SaeS kinase	NA	NA	KAR41_07105	KAR50_05360	KAR53_03075	NA

Biofilm	*bdlA*	NA	NA	KAR41_07485	KAR50_07410	KAR53_07640	NA

a ^T^, type strain. NA, not annotated.

The biofilm dispersion protein BdlA was annotated in *P. fabalis* LMG 26217^T^ (KAR41_07485), *P. fabaria* LMG 24289^T^ (KAR50_07410), and *P. ghanensis* DSM 19935^T^ (KAR53_07640). The *arl*S/*arl*R genes coding for the two-component regulatory system ArlS/ArlR, involved in the regulation of adhesion, autolysis, multidrug resistance, and virulence, were detected in all genomes, while SaeR/SaeS, which is involved in the regulation of virulence factors in staphylococcal strains (24), was predicted in *P. fabalis* LMG 26217^T^, *P. fabaria* LMG 24289^T^, and *P. ghanensis* DSM 19935^T^.

**(iii) Gene clusters for putative bacteriocin production.** The prediction of gene clusters putatively involved in bacteriocin production was performed using BAGEL4. In *P. beninensis* LMG 25373^T^, *P. fabaria* LMG 24289^T^, and *P. ghanensis* DSM 19935^T^, no such cluster could be identified. In *W. diestrammenae* DSM 27940^T^ one area of interest (AOI) for lacticin Q (bacteriocin class II) was predicted (KAR27_07735; aureocin A53 family class IId bacteriocin) (NODE_5:89606.109744). In *P. fabalis* LMG 26217^T^ one AOI for garvicin Q production (KAR41_01340; garvicin Q family class II bacteriocin) was identified, in a putative plasmid comprising a site-specific integrase, one IS6 family transposase, and a *rep* protein (NODE_13:1055.20400). In *W. uvarum* B18NM42^T^ four AOIs were detected. The first AOI (NODE_1:28820.49003) includes a gene coding for a bacteriocin of class II (KAR63_00175; leucocin A/sakacin P family class II bacteriocin). The second AOI (NODE_1:53868.73982) putatively produces one enterocin L50, belonging to the family of leaderless bacteriocins (KAR63_00290). The third AOI (NODE_3:185729.210943) putatively produces gallidermin (KAR63_05910; gallidermin/nisin family lantibiotic), a lantibiotic, bacteriocin-like peptide of type A, while in the fourth AOI (NODE_13:8.10979) there were 3 predicted core peptides annotated as hypothetical proteins (KAR63_03405, KAR63_03425, and KAR63_03420) (see Table S1 in the supplemental material).

**(iv) Antibiotic resistance determinants.** Table 3 shows the presence of several genes coding for unspecific multidrug resistance transporters and proteins in all the strains, with the exception of genes putatively conferring resistance to peptide antibiotics (*mprF*), which were absent in *W. uvarum* B18NM42^T^, and to streptogramin A, virginiamycin M, lincosamide, lincomycin, pleuromutilin, and tiamulin (*expZ*), which were missing in *P. beninensis* LMG 25373^T^ and *W. diestrammenae* DSM 27940^T^. As for genes involved in resistance to specific antibiotic classes, tetracycline resistance proteins were present in *P. beninensis* LMG 25373^T^, *P. fabalis* LMG 26217^T^, *P. fabaria* LMG 24289^T^, and *P. ghanensis* DSM 19935^T^. A fosfomycin resistance gene occurred in *P. fabalis* LMG 26217^T^, *P. fabaria* LMG 24289^T^, and *P. ghanensis* DSM 19935^T^, while a bicyclomycin resistance gene was identified in *W. diestrammenae* DSM 27940^T^ and in *W. uvarum* B18NM42^T^. Furthermore, genes involved in macrolide and bacitracin resistance were found in all the analyzed strains.

**TABLE 3:** Genetic determinants related to antibiotic resistance in *Periweissella* and *Weissella* strains (see “Bioinformatic methods” for details)^*a*^

Antibiotic class	Function	Gene	*P. beninensis* LMG 25373^T^	*W. diestrammenae* DSM 27940^T^	*P. fabalis* LMG 26217^T^	*P. fabaria* LMG 24289^T^	*P. ghanensis* DSM 19935^T^	*W. uvarum* B18NM42^T^
Peptide antibiotic	Bifunctional lysylphosphatidylglycerol flippase/synthetase MprF	*mprF*	KAK10_01195	KAR27_03035	KAR41_07315	KAR50_05085	KAR53_00640	NA

Multidrug	Probable multidrug resistance ABC transporter ATP-binding/permease protein YheH	*yheH*	KAK10_02605	NA	KAR41_06485	KAR50_06245	KAR53_02480	NA

Multidrug	Probable multidrug resistance ABC transporter ATP-binding/permease protein YheI	*yehI*	KAK10_02610	NA	KAR41_06480	KAR50_06250	KAR53_02485	NA

Multidrug	ABC-type bacteriocin transporter/multidrug resistance protein	*lmrA*	NA	KAR27_04580	NA	NA	NA	NA

Bacitracin	Bacitracin transport ATP-binding protein BcrA	*bcrA*	NA	NA	KAR41_05070	KAR50_00960	KAR53_04525	KAR63_05905
	Bacitracin export ATP-binding protein BceA	*bceA*	KAK10_02500	KAR27_06075, KAR27_07950	KAR41_06615	KAR50_06080	KAR53_02335	KAR63_06125
	Bacitracin export permease protein BceB	*bceB*	NA	KAR27_07955	KAR41_06620	NA	NA	NA
	Undecaprenyl-diphosphate phosphatase	*bcrD*	NA	KAR27_00065	KAR41_07965	KAR50_06900	KAR53_09885	KAR63_02685

Macrolide	ATP-binding cassette domain-containing protein	*macB*	KAK10_08270	KAR27_06610	KAR41_01530, KAR41_08365	KAR50_03530, KAR50_05050	KAR53_08780	KAR63_02640, KAR63_03695

Streptogramin A, virginiamycin M, lincosamide, lincomycin, pleuromutilin, tiamulin	ATP-binding cassette domain-containing protein	*expZ*	NA	NA	KAR41_01500, KAR41_06395	KAR50_09285	KAR53_06405	KAR63_03690

Bicyclomycin	Multidrug efflux MFS transporter	*bcr*	NA	KAR27_07875	NA	NA	NA	KAR63_04970

Multidrug	MFS transporter	*stp*	NA	NA	KAR41_04925	KAR50_01130	KAR53_04765	KAR63_00480

Multidrug	MATE family efflux transporter	*mepA*	KAK10_02905	KAR27_02785	KAR41_00820	KAR50_09080	KAR53_00380	NA

Multidrug	Multidrug export protein EmrB	*emrB*	KAK10_07025	KAR27_07200, KAR27_06420	KAR41_04745, KAR41_07660	KAR50_07235, KAR50_01360	KAR53_01010, KAR53_04580	KAR63_04340

Multidrug	Multidrug transporter EmrE	*emrE*	NA	NA	KAR41_06800	KAR50_05895	KAR53_09040	NA

Multidrug	Multidrug efflux MFS transporter EmrY	*emrY*	NA	KAR27_02805	KAR41_07550	KAR50_07345	KAR53_02530	NA
Multidrug	MFS transporter	*mdrP*	NA	KAR27_02725	KAR41_05095	NA	KAR53_04955	KAR63_00455/KAR63_00830

Fosfomycin	HAD family hydrolase	*mupP*	NA	NA	KAR41_07275	KAR50_05205	KAR53_09090	NA

Multidrug	Multidrug efflux SMR transporter	*qacC*	KAK10_04780	NA	NA	NA	NA	KAR63_05790

Multidrug	ABC transporter permease	*Rv1217c*	KAK10_02225	NA	NA	KAR50_08380	NA	NA

Multidrug	ABC transporter ATP-binding protein	*Rv1218c*	KAK10_02230	NA	NA	KAR50_08385, KAR50_06335	KAR53_02610	NA

Tetracycline	Tetracycline resistance protein TetA/multidrug resistance protein Mdt/MFS transporter	*tetA*	KAK10_06035	NA	KAR41_00180	KAR50_01955	KAR53_06840	NA
	TetM/TetW/TetO/TetS family tetracycline resistance ribosomal protection protein	*tetM*	NA	NA	KAR41_07840	KAR50_07020	KAR53_05135	NA
		*tetO*	NA	NA	NA	NA	KAR53_01250	NA

Multidrug	CDP-diacylglycerol–glycerol-3-phosphate 3-phosphatidyltransferase (EC 2.7.8.5)	*pgsA*	KAK10_05885	KAR27_01760	KAR41_03120	KAR50_07900	KAR53_09415	KAR63_04370

Multidrug	Glycerophosphoryl diester phosphodiesterase (EC 3.1.4.46)	*gdpD*	KAK10_06615	KAR27_05880	KAR41_03685, KAR41_08810	KAR50_02600, KAR50_03090	KAR53_00095	KAR63_03110, KAR63_01630

Multidrug	Zinc ABC transporter substrate-binding protein	*ZnuA*	NA	KAR27_02765	NA	NA	NA	KAR63_00515

Multidrug	Periplasmic solute binding protein, ZnuA-like	*ZnuC*	KAK10_06935	KAR27_02770	KAR41_05160	KAR50_00880	KAR53_05015	KAR63_00520

Multidrug	ABC transporter, TroCD-like	*ZnuB*	KAK10_06940	KAR27_02775	KAR41_05155	KAR50_00885	KAR53_05010	KAR63_00525

Multidrug	ABC-transporter extension domain	*YbiT*	KAK10_05380	KAR27_02780	KAR41_08250	KAR50_09450	KAR53_08895	KAR63_00530

a ^T^, type strain; NA, not annotated.

### Phenotypic analysis.

**(i) Assessment of the probiotic and biotechnological potential.** *(a) EPS production.* The exopolysaccharide (EPS) production was investigated by using De Man, Rogosa, and Sharpe (MRS) agar supplemented with either glucose, sucrose, or raffinose. After 48 h of incubation, only the *P. beninensis* type strain showed the presence of clear, ropy, and viscous material on MRS supplemented with sucrose. Negative plates inoculated with the other *Weissella* and *Periweissella* type strains were further incubated for additional 24 h, but still no EPS production could be detected.

*(b) Antimicrobial activity.* The petri dishes were observed after 48 and 72 h of incubation. No inhibitory activity against Escherichia coli DSM 30083, Bacillus megaterium F6, and Listeria monocytogenes ATCC 19115 could be detected.

*(c) Survival under simulated gastrointestinal conditions.* Results of the evaluation of the viability of the strains under *in vitro* simulated gastrointestinal conditions are reported in Table 4. Lacticaseibacillus rhamnosus LGG was used as a positive control, and although a significant decrease (*P *< 0.005, Bonferroni corrected) compared to the initial cell density was observed under all pH conditions throughout the incubation time, it was never below 8 log_10_ CFU/mL. Except for the *P. beninensis* and *P. fabalis* type strains, microbial numbers of all the other type strains drastically decreased (*P *< 0.005, Bonferroni corrected) by ca. 8 log_10_ cycles, reaching numbers below 2 log_10_ CFU/mL (Table 4) after incubation at pH 2.0 for 360 min. The *P. fabalis* type strain reached a count of 3.28 ± 0.22 log_10_ CFU/mL, whereas the *P. beninensis* type strain, being the least affected by the simulated gastric condition at pH 2.0 (*P *< 0.00238, Bonferroni corrected), remained vital up to 7 log_10_ CFU/mL. A slightly higher survival was registered at pH 3.0 (Table 4), especially for the *W. uvarum* type strain, which retained a bacterial count of 8 log_10_ CFU/mL after 6 h, while the *P. beninensis* type strain showed a trend similar to that observed for pH 2.0. The slight decrease in viability observed in simulated gastric fluid (SGF) at pH 8.0 for all the strains but the *W. fabalis* type strain suggested that it might be largely caused by the low pH rather than the effect of the rest of the constituents of the SGF. Similarly, the addition of reconstituted skimmed milk (RSM) to the sample mixture at pH 2.0 (Table 4) increased the survival of all the strains, which never reached densities below 6 log_10_ cycles after 360 min, which most probably resulted from the protective effect of the matrix and the buffer effect on the pH.

**TABLE 4:** Survival of the *Periweissella* and *Weissella* type strains under simulated gastric conditions (0 to 180 min) at pH 2.0, 3.0, 8.0, and 2.0 with addition of reconstituted skimmed milk (RSM; 11%, wt/vol), and further intestinal digestion (180 to 360 min) at pH 8.0^*a*^

Strain	pH	Viable counts (log CFU/mL) in fluid at time				
		Simulated gastric fluid			Simulated intestinal fluid	
		0 min	90 min	180 min	270 min	360 min
L. rhamnosus LGG	pH2	9.48 ± 0.05 Aa	9.37 ± 0.02 Aa	8.91 ± 0.06 Ab	8.23 ± 0.04 Ac	7.97 ± 0.08 Ad
	pH3	9.53 ± 0.05 Aa	9.49 ± 0.05 Aa	9.09 ± 0.05 Ab	8.48 ± 0.04 Ac	8.02 ± 0.04 Ad
	pH8	9.42 ± 0.04 Aa	9.38 ± 0.08 Aa	9.24 ± 0.07 Aab	9.01 ± 0.02 Abc	8.85 ± 0.06 Ac
	pH2+RSM	9.47 ± 0.06 Aa	9.52 ± 0.07 Aa	9.48 ± 0.07 Aa	9.18 ± 0.07 Ab	9.11 ± 0.04 Ab

*P. beninensis* LMG 25373^T^	pH2	8.93 ± 0.04 BCa	8.77 ± 0.08 Aa	7.92 ± 0.14 Bb	7.89 ± 0.28 Ab	6.64 ± 0.28 Bc
	pH3	8.93 ± 0.04 Ca	8.72 ± 0.04 Ca	8.18 ± 0.04 Cb	8.19 ± 0.06 Bb	7.07 ± 0.05 Cc
	pH8	8.93 ± 0.04 Ca	8.15 ± 0.08 Eb	7.07 ± 0.04 Ec	6.92 ± 0.05 Dc	6.42 ± 0.04 Bd
	pH2+RSM	8.93 ± 0.04 Ca	8.52 ± 0.04 Db	8.28 ± 0.04 Ec	7.35 ± 0.03 Dd	6.51 ± 0.03 De

*W. diestrammenae* DSM 27940^T^	pH2	8.98 ± 0.03 Ba	6.76 ± 0.02 Db	3.93 ± 0.05 Ec	1.02 ± 0.01 Dd	<1 CFU/mL
	pH3	9.1 ± 0.03 Ba	9.13 ± 0.06 Ba	9.06 ± 0.03 Aa	4.03 ± 0.02 Fb	3.63 ± 0.03 Ec
	pH8	9.11 ± 0.03 Ba	9.13 ± 0.03 Ba	9.13 ± 0.04 Aa	5.49 ± 0.03 Eb	5.37 ± 0.03 Db
	pH2+RSM	9.11 ± 0.03 Bab	9.13 ± 0.06 Ba	8.94 ± 0.03 Cb	8.1 ± 0.03 Bc	8.08 ± 0.03 Bc

*P. ghanensis* DSM 19935^T^	pH2	8.5 ± 0.04 Ea	4.8 ± 0.1 Eb	2.4 ± 0.01 Fc	1.2 ± 0.01 Dd	<1 CFU/mL
	pH3	8.98 ± 0.05 BCa	8.68 ± 0.05 Cb	7.78 ± 0.04 Dc	5.63 ± 0.03 Dd	5.46 ± 0.04 Dd
	pH8	9.17 ± 0.0 Ba	8.71 ± 0.05 Cb	8.35 ± 0.06 Cc	6.16 ± 0.04 Dd	6.1 ± 0.08 Cd
	pH2+RSM	9.33 ± 0.05 Aa	9.27 ± 0.05 Ba	9.26 ± 0.05 Ba	9.26 ± 0.06 Aa	9.25 ± 0.07 Aa

*P. fabalis* LMG 26217^T^	pH2	8.52 ± 0.05 Ea	8.52 ± 0.06 Ca	6.32 ± 0.08 Cb	4.48 ± 0.21 Bc	3.28 ± 0.22 Cd
	pH3	8.56 ± 0.05 Ea	7.74 ± 0.07 Db	6.67 ± 0.04 Ec	4.2 ± 0.05 Ed	3.34 ± 0.05 Fe
	pH8	8.47 ± 0.05 Ea	8.07 ± 0.04 Eb	5.39 ± 0.03 Fc	5.2 ± 0.05 Fc	4.18 ± 0.04 Ed
	pH2+RSM	8.42 ± 0.05 Ea	7.58 ± 0.04 Eb	7.61 ± 0.06 Fb	7.53 ± 0.05 Cb	6.59 ± 0.04 Dc

*P. fabaria* LMG 25373^T^	pH2	8.73 ± 0.05 Da	4.57 ± 0.03 Eb	2.32 ± 0.04 Fc	<1 CFU/mL	<1 CFU/mL
	pH3	8.76 ± 0.05 Da	5.32 ± 0.03 Eb	3.01 ± 0.02 Fc	1.03 ± 0.01 Gd	<1 CFU/mL
	pH8	8.68 ± 0.05 Da	8.44 ± 0.05 Db	7.81 ± 0.05 Dc	6.43 ± 0.04 Cd	5.97 ± 0.04 Ce
	pH2+RSM	8.64 ± 0.05 Da	8.56 ± 0.05 Da	8.29 ± 0.08 Eb	7.51 ± 0.05 Cc	7.29 ± 0.05 Cb

*W. uvarum* B18NM42^T^	pH2	8.8 ± 0.05 CDa	6.83 ± 0.08 Db	5.65 ± 0.13 Dc	1.85 ± 0.02 Cd	1.74 ± 0.05 Dd
	pH3	8.67 ± 0.05 DEa	8.79 ± 0.09 Ca	8.82 ± 0.03 Ba	7.86 ± 0.04 Cb	7.84 ± 0.05 Bb
	pH8	8.74 ± 0.05 Da	8.74 ± 0.06 Ca	8.73 ± 0.06 Ba	8.75 ± 0.07 Ba	8.75 ± 0.08 Aa
	pH2+RSM	8.79 ± 0.05 Ca	8.79 ± 0.06 Ca	8.67 ± 0.06 Da	7.97 ± 0.06Bb	7.98 ± 0.06 Bb

a *Lacticaseibacillus rhamnosus* LGG was used as a positive control. ^T^, type strain. Values are the mean for three replicates ± standard deviation. Different lowercase letters in the same row mean significant differences at a *P* value of <0.005 (*P*, Bonferroni corrected). Different uppercase letters in the same column, within the same pH condition, mean significant differences at a *P* value of <0.00238 (*P*, Bonferroni corrected).

**FIG 1:**
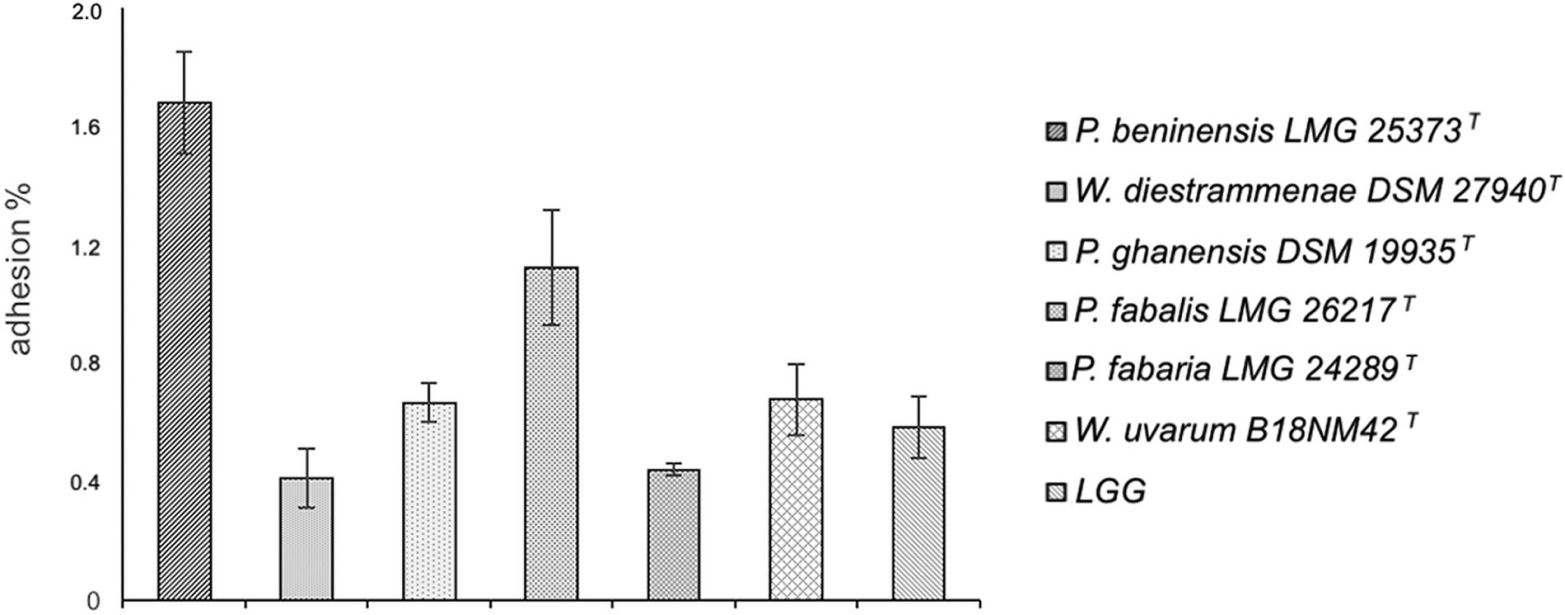
Adhesion capacity of *Weissella* and *Periweissella* strains. The capacity for adhesion to Caco-2 cells was expressed as percentage of the adhered bacterial cell per the initial bacterial density. L. rhamnosus GG was included as a control. Each adhesion assay was conducted three times. The error bars indicate standard errors. No statistically significant differences were observed (*P* > 0.00238, Bonferroni corrected).

*(d) Adhesion.* Results for the adhesion capacity of the two *Weissella* and four *Periweissella* type strains for Caco-2 cells are shown in Fig. 1. *Lacticaseibacillus rhamnosus* LGG, which is a probiotic strain well known for its high capacity of binding to intestinal cells, was used as a positive control. The *P. fabalis* type strain showed an adhesion of 1.2%, while *W. diestrammenae*, *W. uvarum*, *P. ghanensis*, and *P. fabaria* type strains had an adhesive capacity similar to that of LGG (0.6%), ranging from 0.4 to 0.7%. *P. beninensis* LMG 25373^T^ showed a certain higher adhesion capacity, with an adhesion of 1.7%, meaning that, starting from 10^8^ CFU, almost 1.6 × 10^6^ CFU were able to adhere to the Caco-2 cells, but no statistically significant differences from L. rhamnosus LGG or the other tested strains could be inferred based on the results obtained here (*P* > 0.00238, Bonferroni corrected).

**FIG 2:**
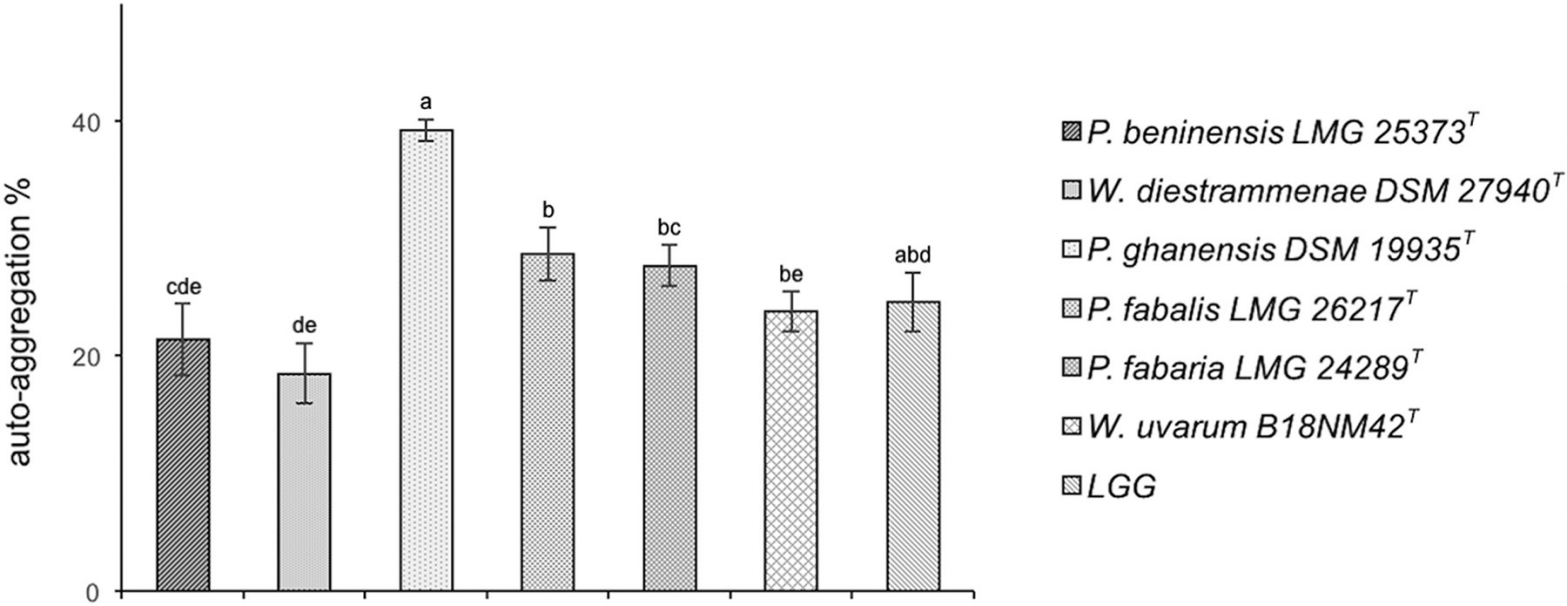
Autoaggregation capacity of *Weissella* and *Periweissella* strains. L. rhamnosus GG was included as a control. Each assay was conducted three times. The error bars indicate standard errors. Different letters mean statistically significant differences at a *P* value of <0.00238 (*P*, Bonferroni corrected).

*(e) Autoaggregation.* Results of the autoaggregation assays are shown in Fig. 2. A certain degree of autoaggregation was already observed after 1 h of incubation in all the strains tested in this study (from 4.5% to 29.3%; data not shown) and increased continually with time. At 4 h, the highest percentage of autoaggregation was achieved, ranging from 18.5 to 39.2%. The *P. ghanensis* type strain showed the greatest capacity to autoaggregate after 4 h (39.2%) compared to the other tested *Weissella* and *Periweissella* type strains (*P* < 0.00238, Bonferroni corrected), which showed an autoaggregation ability ranging from 18.5% (*W. diestrammenae* type strain) to 28.7% (*P. fabalis* type strain).

**FIG 3:**
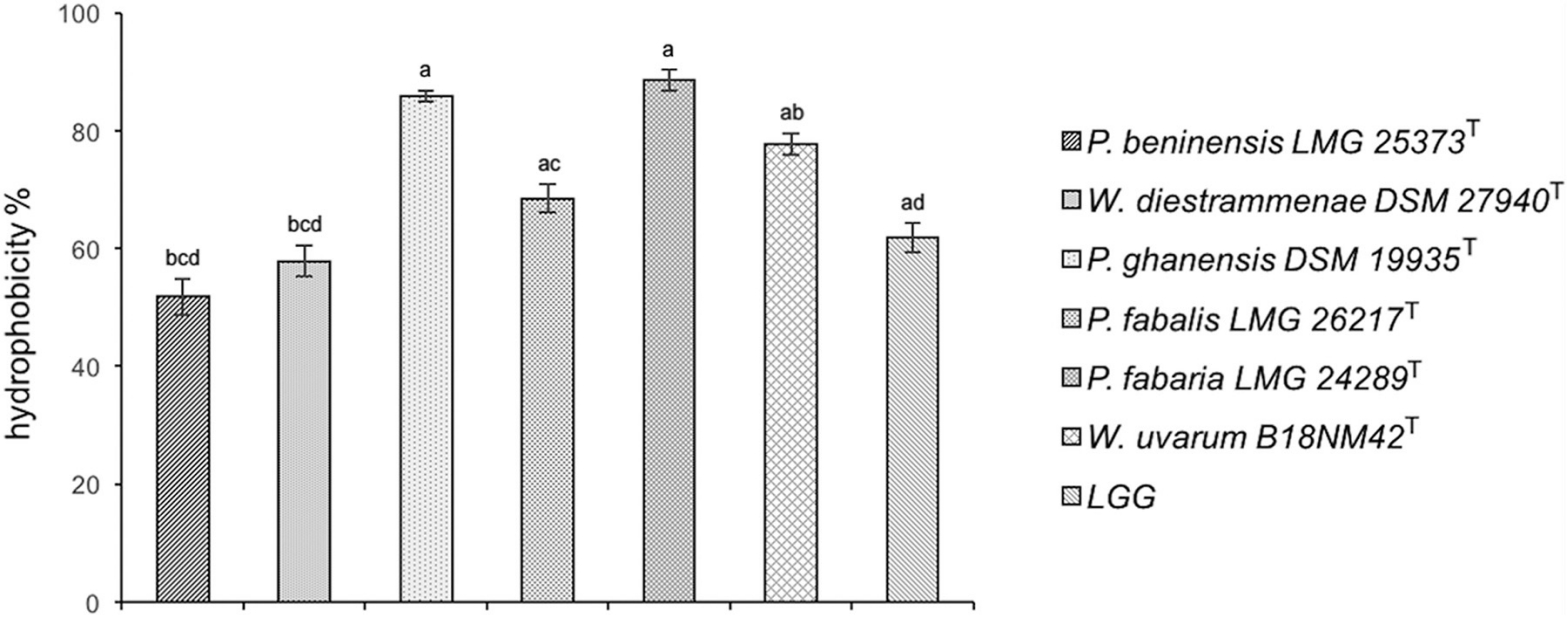
Cell surface hydrophobicity of *Weissella* and *Periweissella* strains. L. rhamnosus GG was included as a control. Each assay was conducted three times. The error bars indicate standard errors. Different letters mean statistically significant differences at a *P* value of <0.00238 (*P*, Bonferroni corrected).

*(f) Cell surface hydrophobicity.* Results of cell surface hydrophobicity tests are shown in Fig. 3. *P. fabaria* LMG 24289^T^ and *P. ghanensis* DSM 19935^T^ displayed the highest values of cell hydrophobicity, of 88.5 and 85.8%, respectively, which were significantly different (*P* < 0.00238, Bonferroni corrected) from those displayed by *P. beninensis* LMG 25373^T^ (51.8%) and *W. diestrammenae* DSM 27940^T^ (57.8%), which showed the lowest percentages.

**(ii) Safety assessment.** *(a) Hemolytic assay.* No clear or greenish zone around bacterial colonies was observed, indicating that all the *Weissella* and *Periweissella* strains tested were nonhemolytic.

*(b) Antibiotic resistance.* The phenotypic susceptibility of strains to antimicrobials is shown in Table 5. Vancomycin resistance was observed for all strains, with the exception of *P. beninensis* LMG 25373^T^. *W. diestrammenae* DSM 27940^T^ was also resistant to streptomycin and, together with *P. fabaria* LMG 24289^T^, showed resistance to gentamicin and kanamycin as well. *P. ghanensis* DSM 19935^T^ and *W. uvarum* B18NM42^T^ were resistant to erythromycin, while *P. fabalis* LMG 26217^T^, *P. ghanensis* DSM 19935^T^, *W. uvarum* B18NM42^T^, and *P. fabaria* LMG 24289^T^ showed resistance to chloramphenicol.

**TABLE 5:** Antimicrobial susceptibilities of *Periweissella* and *Weissella* strains^*a*^

Antibiotic	MIC (mg/L) for strain:					
	*P. beninensis* LMG 25373^T^	*W. diestrammenae* DSM 27940^T^	*P. fabalis* LMG 26217^T^	*P. ghanensis* DSM 19935^T^	*W. uvarum* B18NM42^T^	*P. fabaria* LMG 24289^T^
Ampicillin	1	1	1	1	2.5	2.5
Vancomycin	20	R	R	R	R	R
Gentamicin	10	R	40	40	40	R
Kanamycin	80	R	80	160	160	R
Streptomycin	20	R	80	40	20	80
Erythromycin	2.5	2.5	2.5	R	R	5
Clindamycin	0.5	0.5	0.5	0.5	0.5	0.25
Tetracycline	5	10	20	20	5	10
Chloramphenicol	20	20	R	R	R	R

a MICs of ampicillin, vancomycin, gentamicin, kanamycin, streptomycin, erythromycin, clindamycin, tetracycline, and chloramphenicol were assessed using cutoff concentrations as described by the EFSA Panel on Additives and Products or Substances used in Animal Feed (FEEDAP) panel (62) as well as using concentrations 2.5-, 5-, and 10-fold higher than cutoff values. ^T^, type strain. Each assay was conducted three times. R, resistant, strain not inhibited at a concentration >10-fold the cutoff value described by the FEEDAP panel (62).

## DISCUSSION

In this work, we analyzed the probiotic potential of six type strains of *Weissella* and *Periweissella* spp. by combining the *in silico* prediction of desirable and undesirable features, such as the presence of probiotic genes on the one side, and the presence of antimicrobial resistance genes or virulence determinants on the other, with a comprehensive *in vitro* evaluation of the attributes that are mandatory for good probiotic candidates, such as the capacity for adhesion to the epithelial cells, autoaggregation capacity, hydrophobicity, and survival of the gastrointestinal tract.

We performed a homology-based analysis to verify the presence of genes putatively involved with the functional and probiotic capacities of *Weissella* and *Periweissella* type strains, whose genomes were recently sequenced and reported by Fanelli et al. (2); the analysis was based on the genetic screening previously performed by Turpin et al. (19). The set of genes evaluated include those involved in bile salt tolerance, pH survival, biogenic amine synthesis, riboflavin synthesis, folate synthesis, and starch metabolism. The housekeeping gene *groEL*, which was found to be present in all the strains, codes for a 60-kDa heat shock protein (chaperonin 60), which is reported to be more abundant in acid-stressed LAB and thus is thought to play an important role in their survival and adaptation to low pH (25). The *dltD* gene (encoding a d-alanine transfer protein) was found in all but the *P. beninensis* type strain, while the *La57* gene (encoding an amino acid antiporter) was found only in the *W. diestrammenae*, *P. fabalis*, and *W. uvarum* type strains. Both these genes are also involved in low-pH survival (19). All the strains and all but the *P. beninensis* type strain harbored *LBA1446* (encoding a multidrug resistance protein) and *LBA1679* (encoding an ABC transporter), respectively, while only the *P. ghanensis*, *W. uvarum*, and *P. fabalis* type strains harbored the *LBA1432* gene (encoding a hypothetical protein). All these genes are involved in the bile salt tolerance (19).

Riboflavin (vitamin B_2_) is the precursor of the coenzymes flavin mononucleotide (FMN) and flavin adenine dinucleotide (FAD), essential carriers in the redox reaction of cell metabolism (26). In addition to their roles in biochemical reaction, riboflavins are also involved in host-microbe signaling and quorum sensing (27, 28). The LAB ability to synthesize riboflavin has been exploited to obtain fermented bioenriched foods (29). Biosynthesis of riboflavin from GTP and ribulose-5-phosphate requires the presence of a complete *rib* operon (*ribD*, *ribE*, *ribAB*, and *ribH*); *ribCF* encodes a bifunctional riboflavin kinase/FMN adenylyltransferase, which converts riboflavin into FMN and FAD, while *ribU* encodes a transporter for the uptake of the preformed riboflavin. *ribCF* and *ribU* are located in a separate genomic locus with respect to the operon, as described in Bifidobacterium longum by Solopova et al. (30). The complete set of riboflavin biosynthetic genes was retrieved only in *P. fabalis* LMG 26217^T^, *P. fabaria* LMG 24289^T^, and *P. ghanensis* DSM 19935^T^; this finding suggests their putative capability of producing these compounds and of being used as biofortification agents, exploiting the possibility to increase the riboflavin biosynthesis through fermentative processes (31). All strains with the exception of *W. diestrammenae* DSM 27940^T^ and *P. beninensis* LMG 25373^T^ harbored the *folP* and *folK* genes, encoding the dihydropteroate synthase and 2-amino-4-hydroxy-6-hydroxymethyldihydropteridine diphosphokinase, respectively; both genes are involved in folate (vitamin B_9_) synthesis (19), leading to the hypothesis that these strains may exert the probiotic function of accomplishing folate biosynthesis *in vivo* within the colon, allowing treatment or prevention of low-folate conditions. Indeed, while folate dietary vitamins are largely absorbed in the small intestine (32), folates synthesized by probiotics are primarily absorbed in the colon (33).

With the exception of the *W. uvarum* type strain, all the remaining type strains analyzed harbored the linoleate isomerase (annotated by NCBI as oleate hydratase). In LAB, this protein catalyzes the production of conjugated linoleic acid (CLA) from alpha-linoleic acid. CLA exerts health-associated benefits including anticancer, antioxidant, antiobesity, and antiatherosclerosis activities, as well as improved immune system function and normalization of impaired glucose tolerance in animals and humans (34).

In the predicted proteomes of *P. fabalis* LMG 26217^T^, *P. fabaria* LMG 24289^T^, and *W. uvarum* B18NM42^T^, we identified homologs of the oligo-1,6-glucosidase and the α-glucosidase encoded by the *malL* and *agl* genes, respectively. The latter enzyme hydrolyzes only the α-1,6 linkage in starch, glycogen, and the oligosaccharides derived from them, to produce sugars with an α-configuration, while it cannot hydrolyze the α-1,4 linkage. The presence of these genes led to the hypothesis of the ability of these strains to allow the entry of starch degradation products, such as maltose, into the glycolytic pathway (19, 35). Since not all the starch introduced in the diet is hydrolyzed by human enzymes, being partially available as an energy source for the gut microbiota (36), glucosidases might be of utmost importance for probiotics, allowing them to be competitive in the human gastrointestinal (37) and genitourinary (38) tracts. In addition, the starch fermentation by gut microbiota, including probiotics, produces as beneficial products the short-chain fatty acids (SCFAs) which are documented to have a relevant role in healthy aging and exploit anti-inflammatory and antiproliferative effects (39, 40).

Based on the assessment of the survival under *in vitro* simulated gastrointestinal conditions, the *P. beninensis* type strain showed the best tolerance of both acidic and bile conditions, exhibiting a trend more similar to that of L. rhamnosus LGG, whereas the other type strains revealed a resistance ranging from moderate to good, according to the classification originally described by Chateau et al. (41) and recently used by Sadeghi et al. (42). Such tolerance is consistent with the low-pH conditions occurring during the fermentation of cassava, from which *P. beninensis* LMG 25373^T^ had been first isolated (7). The increased survival rate for all the strains when suspended in reconstituted skimmed milk leads to the hypothesis that milk and (most likely) milk-derived matrices could serve as good carriers of these potential probiotics.

The capability of probiotics to adhere to epithelial cells and mucosal surfaces without being eliminated from the large intestine by peristalsis is an important property that allows colonization of the human intestinal tract (43), increasing their persistence while performing their beneficial effects. In addition, the attachment of probiotics to the intestinal mucosa is essential to exert any potential protective role against enteropathogens through competition for host cell binding sites. Although no statistically significant difference could be inferred, based on our results, the *P. beninensis* type strain showed the highest adherence ability (1.7% adhesion), followed by the *P. fabalis* type strain (1.2% adhesion), while all the other type strains revealed an adherence ability similar to that of the probiotic strain L. rhamnosus LGG.

The production of EPS facilitates gut colonization, thus representing a trait for probiotic bacterial evaluation (44). The EPS production influences the biochemical properties of the cell, including hydrophobicity and surface charge; EPS stabilizes microbial biofilm, enhancing bacterial adhesion and aggregation and increasing the tolerance to environmental stress (45). The consumption of sucrose and raffinose by *P. beninensis* LMG 25373^T^ was previously demonstrated (2), and only this strain was able to produce EPS in MRS agar supplemented with sucrose.

Another essential property of probiotics is the ability to self-aggregate; this property is the first step necessary for colonization, recognition, communication, and survival of bacteria, and allows them to reach a high cell density in the gastrointestinal tract, favoring the adhesion to intestinal epithelial cells and mucosal surface. Furthermore, autoaggregated cells may form a barrier that prevents colonization by pathogenic microorganisms (46, 47, 48). Auto-aggregation percentages increased over the time for all tested strains (data not shown) and after 4 h of incubation reached values between 18.5% (*W. diestrammenae* DSM 27940^T^) and 39.2% (*P. ghanensis* DSM 19935^T^). Our data agreed with those reported by other authors which assessed the LGG autoaggregation capacity at 23% after 5 h of incubation (49, 50) and a range of autoaggregation capacities of *Lactobacillus* probiotic strains between 24 and 40% (51).

Hydrophobicity is among the most important features for cell surface properties of probiotics, and some authors reported its correlation with the ability of probiotics to adhere to epithelial cells (52–54). Although our strains revealed a high hydrophobicity, ranging from ca. 52% to 86% and in line with those reported for *Lactobacillus* probiotic strains (51), a higher hydrophobicity did not result in an increased adhesion ability of the relevant strains. This is not surprising, since the properties of cell adhesion are influenced by other factors such as pH, temperature, and composition of the culture medium, so that other studies also did not find any correlation between hydrophobicity and adhesion (55–58).

The *in silico* approach for the risk assessment of probiotic lactic acid bacteria was recently discussed by Peng et al. (20), who defined the WGS-based informatics analyses as an ideal and cost-effective approach for preliminary risk evaluation. This initial screening, however, needs to be coupled with *in vitro* assays in order to avoid the exclusion of potential probiotic strains based only on the presence of undesirable genes that may be unexpressed or not functional under the conditions or in the environments in which the probiotics should exploit their function. Indeed, our data demonstrated that, although all type strains harbor hemolysin genes, the hemolytic assay we performed gave negative results for all the strains tested. The absence of acquired and transferable antibiotic resistance is an ideal requisite that probiotics should have (59). Nevertheless, antibiotic resistance determinants have been reported in probiotic bacteria used in dairy products, starter cultures, and probiotic foods as well as dietary supplements (60). With due caution, this may represent an exploitable feature if probiotics and antibiotics are coadministered to prevent gastrointestinal disorders due to the antibiotic treatment (59). We should underline that antibiotic resistance studies on *Weissella* and *Periweissella* are limited (16, 61), and to date, no reference cutoff values are established to determine their antibiotic resistance. Here, we assessed the MIC values of the six *Weissella* and *Periweissella* type strains analyzed, evaluating antibiotic concentrations even 10-fold higher than the ones reported by the FEEDAP panel in the guidance on the assessment of bacterial susceptibility to antimicrobials of human and veterinary importance (62). For the majority of these type strains, we found MIC values higher than the general cutoff values set by the Panel for “other Gram-positive bacteria” (62). However, it is worth noting that *P. beninensis* LMG 25373^T^ showed, overall, lower MIC values than the other *Weissella* and *Periweissella* type strains tested in the present work and was found to harbor fewer genes involved in resistance to specific antibiotic classes (*bceA*, *macB*, *tetA*, *mprF*) than the other type strains analyzed in this study.

Based on our results, all strains, with the exception of *P. beninensis* LMG 25373^T^, were classified as resistant to vancomycin, although we did not detect any vancomycin resistance genes in all the analyzed type strains. This antibiotic interferes with precursors of peptidoglycan synthesis, binding to d-Ala/d-Ala dipeptide and inhibiting polymerization (15), thus inhibiting cell wall biosynthesis. Resistance to vancomycin was noticed within the *Weissella* genus, as reported by Fhoula et al. (63) and Abriouel et al. (11), and, due to this resistance (besides their unusual Gram stain morphology), *Weissella* spp. have been often confused with *Lactobacillus* spp. or *Lactobacillus*-like organisms (1). Such resistance may be considered “intrinsic” (16), since in *Weissella* spp. the terminal d-Ala is absent while d-lactate or d-Ser occurs in its place; thus, they do not bind to vancomycin (15). This explains the generally high MIC values for this antibiotic that have been reported also by other authors (even higher than 128 mg/L) (16, 64). With the exception of *P. beninensis* LMG 25373^T^ and *W. diestrammenae* DSM 27940^T^, all the analyzed type strains were classified as resistant to chloramphenicol. Similar to our findings, high MIC values for chloramphenicol (even 16 and 32 mg/L) were also reported for some Weissella confusa strains in previous studies (63, 65). Considering this, the presence of specific chloramphenicol resistance genes was suggested in *Weissella* spp. (65). Nonetheless, the presence of the *cat* gene coding for a chloramphenicol acetyltransferase, which is the most common chloramphenicol resistance gene in LAB (64), was not detected in chloramphenicol-resistant *Weissella* strains (63), in line with our results. In light of this, and as proposed for the related genus *Leuconostoc* (64), the possibility of an intrinsic resistance to chloramphenicol in *Weissella* spp. has been postulated (63), indicating that horizontal gene transfer of such resistance to other bacteria would not occur. Resistance to aminoglycosides was shown by *W. diestrammenae* DSM 27940^T^ (resistant to gentamicin, kanamycin, and streptomycin) and *P. fabaria* LMG 24289^T^ (resistant to gentamicin and kanamycin). The most prevalent mechanism of aminoglycoside resistance in bacteria is the enzymatic modification of aminoglycosides by the activity of three families of modifying enzymes: phosphotransferases, acetyltransferases, and nucleotidyltransferases. Although resistance to aminoglycosides, such as gentamicin, was already reported in *Weissella* species (17, 18), to our knowledge the genetic determinants responsible for this resistance have never been detected in the *Weissella* and *Periweissella* genera. According to our results, *P. ghanensis* DSM 19935^T^ and *W. uvarum* B18NM42^T^ were classified as resistant to erythromycin, a macrolide antibiotic. Although the *mef*(A/E) gene was previously described as an erythromycin resistance determinant, found in the Weissella cibaria SMA25 strain (16), all our analyzed type strains harbored the macrolide resistance *macB* gene. This gene was recently identified also in the genome of *W. cibaria* UTNGt21O (61), a strain with potentially probiotic and biotechnological features. It should be considered that the presence of genes coding for multidrug resistance proteins and transporters, even in the absence of specific resistance genes, may contribute to the resistance and the MIC values reported for the tested antibiotics in the present study. Based on our analyses, such nonspecific mechanisms may, overall, play a certain role in the ability of *Weissella* and *Periweissella* to resist antibiotics.

All genomes were found to harbor genes such as the *cvb* and *arlS*/*arlR* genes, while SaeR/SaeS and BdlA were predicted in *P. fabalis* LMG 26217^T^, *P. fabaria* LMG 24289^T^, and *P. ghanensis* DSM 19935^T^. However, the actual contribution to virulence of these genes has been demonstrated in species belonging to other genera. Usually, the functionality of virulence genes is linked to the genomic contexts they are associated with and, in some cases, to the presence of antimicrobial resistance genes (66). The location downstream from some active promoter region, on the chromosome or in mobile elements, can influence the expression and transfer of these determinants among bacteria. In addition, many environment- and host-related factors may modulate the virulence. With this regard, for instance, the probiotic Escherichia coli Nissle 1917 has been found to harbor a genome highly similar to that of a pathogenic E. coli strain causing urinary tract infections (67). However, the long history of safe use of this strain leads to the assumption that the presence of virulence genes in itself does not necessarily imply a safety issue, since it has to be interpreted within the genomic context they are identified in (68). Thus, prior to excluding the use of these *Weissella* strains as probiotics, the actual contribution to virulence of these genes in these strains as well as in other weissellas has to be demonstrated, evaluating their expression and their interaction with other genes.

Polyamines, including putrescine, spermidine, spermine, and cadaverine, are cationic molecules derived from amino acids which, despite their important physiological activities, may cause adverse toxicological effects and are commonly detected in fermented foods, due to the activity of decarboxylases of fermenting bacteria (69, 70). The potential ability to produce biogenic amines was evaluated based on the presence of decarboxylase genes and *spe* genes involved in putrescine and spermidine production, well characterized in E. coli (71). Although all six type strains harbor genes for the conversion from arginine to ornithine (*arcA* coding for the arginine deaminase, *argF* coding for the ornithine carbamoyltransferase, and the *arcC* gene coding for the carbamate kinase), and the genes coding for the putrescine/spermidine main transport systems, they all lacked the ornithine decarboxylase and the agmatinase genes needed for the pathway to be functional.

The prediction of gene clusters putatively involved in bacteriocin production showed the presence of one area of interest in the *W. diestrammenae* and *P. fabalis* type strains, while four areas of interest were found in the *W. uvarum* type strain. Conversely, the evaluation of antimicrobial production in MRS supernatant by using the agar-well diffusion assay did not show any inhibition. The production of bacteriocin by LAB is a complex topic in which different variables are involved. Some conditions, such as medium composition, gaseous environment, pH, temperature, and growth kinetics of bacteria, might impact the biosynthesis of bacteriocins. Moreover, the best cell growth rate does not necessarily reflect the highest bacteriocin production rate (72–74). Indeed, the bacteriocin production was reported as higher in the exponential phase and usually inhibited during the stationary phase, while suboptimal growth conditions seem to be related to higher bacteriocin production (72). In addition, Chanos and Mygind (75) explained how the presence of competition between LAB and other microorganisms can induce the production of bacteriocins, which generally does not occur under optimal laboratory circumstances. Thus, the ability for bacteriocin production by the *W. diestrammenae*, *P. fabalis*, and *W. uvarum* type strains could probably occur under different growth conditions than the one tested in this work.

Considering both the probiotic and safety assessments of the six type strains, it can be concluded that *P. beninensis* LMG 25373^T^ is a safe potential probiotic strain. This strain in a previous study (2) showed the widest consumption of carbon sources. Indeed, among the six type strains tested, *P. beninensis* LMG 25373^T^ was the only one capable of using d-galactose (indeed, it was found to harbor the *galA* gene, encoding α-galactosidase [EC 3.2.1.22] [KAK10_01740], which hydrolyzes α-1,6-galactoside linkages found in sugars such as raffinose, melibiose, and stachyose and branched polysaccharides like galactomannans and galactoglucomannans), α-d-lactose, lactulose, d-melibiose, β-methyl-d-galactoside, d-raffinose, sucrose, pyruvic acid methyl ester, and UMP (2).

The ability of the *P. beninensis* type strain to metabolize nondigestible sugars, such as oligosaccharides of the raffinose family and galactomannans, is a feature common to several probiotic bacteria (2). Recently, the potential of raffinose as a prebiotic, i.e., a dietary fiber component that cannot be digested by the human gastrointestinal tract but can be selectively fermented by bacteria in the gastrointestinal tract, has been highlighted. Raffinose can increase the growth of LAB and production of short-chain fatty acids (SCFAs), reduce constipation, inhibit the formation of putrefactive compounds from protein, suppress the growth of pathogenic bacteria, and reduce the risk of cardiovascular diseases (76–80). In addition, stachyose can promote intestinal peristalsis, accelerates the excretion of pathogens and toxins, and favorably modulates the microbiota composition of the human and animal gut. This promotes the formation of the dominant bacteria in the digestive tract and inhibits the production of spoilage bacteria, besides alleviating dextran sulfate sodium-induced acute colitis in mice (81–83). Given their potential as prebiotics, raffinose or stachyose could be used in combination with the *P. beninensis* type strain to produce functional food products.

By this preliminary probiotic evaluation, we demonstrated that especially *P. fabalis* LMG 26217^T^, *P. fabaria* LMG 24289^T^, *P. ghanensis* DSM 19935^T^, *W. uvarum* B18NM42^T^, and *P. beninensis* LMG 25373^T^ expressed tolerance to low pH, a wide carbohydrate metabolic capacity, as reported by Fanelli et al. (2), a potential capability of producing vitamins, and the *in vitro* capacity to adhere to colonic epithelial cells. Although the *in vivo* evaluation has to confirm these potentialities, these features suggest the putative colonization and persistence in the gut environment. The antibiotic resistance profiles, the lack of pathogenic traits, and the biotechnological potential shown by our studies indicate, preliminarily, that these strains, and especially *P. beninensis* LMG 25373^T^, represent good candidates as starter cultures or as potential probiotics for application in food industries, although *in vivo* validation and investigation of health effects have to be performed.

## MATERIALS AND METHODS

### Strain information and culture conditions.

*P. beninensis* LMG 25373^T^, *W. diestrammenae* DSM 27940^T^, *P. fabalis* LMG 26217^T^, *P. fabaria* LMG 24289^T^, *P. ghanensis* DSM 19935^T^, and L. rhamnosus GG (LGG, ATCC 53103) and Leuconostoc pseudomesenteroides 20193^T^, used as a reference strain, were purchased from the Leibniz Institute DSMZ-German Collection of Microorganisms and Cell Culture (DSMZ, Germany) and from the Belgian Coordinated Collection of Microorganisms (BCCM/LMG; Belgium); the *W. uvarum* B18NM42 type strain (DSM 28060^T^) was kindly provided by Aspasia Nisiotou from the Institute of Technology of Agricultural Products, Hellenic Agricultural Organization Lycovrissi, Greece. The strains were grown as described by Fusco et al. (84). The purity of each strain was confirmed by streaking on De Man, Rogosa, and Sharpe (MRS; Oxoid, Italy) agar plates and by microscopic observation. Strains were maintained at −80°C as pure stock cultures in MRS broth (Oxoid, Italy) supplemented with 30% (vol/vol) glycerol. Strains were routinely grown on De Man, Rogosa, and Sharpe (MRS; Oxoid, Italy) broth at 30°C under aerobic static conditions unless specified otherwise.

### Bioinformatic methods.

Whole-genome sequencing and *de novo* assembly of the six type strains of *Weissella* and *Periweissella* spp. were performed as described in the work of Fanelli et al. (2). The evaluation of the quality of the assemblies was described in the work of Fanelli et al. (2).

*Weissella* and *Periweissella* type strains proteomes were predicted by using the Prokaryotic Genome Annotation Pipeline (85). All the protein sequences used in this study were retrieved from GenBank (NCBI). The homology-based relationship of *Weissella* and *Periweissella* proteomes with selected proteins was determined by the BLASTP algorithm on the NCBI site (http://blast.ncbi.nlm.nih.gov/Blast.cgi). Virulence determinants were identified by homology-based analysis, searching the *Weissella* and *Periweissella* type strain proteomes against the full data set of the Virulence Factor Database (VFDB) (86) as well as against potential virulence genes of *Weissella* species described by Abriouel et al. (11), with a cutoff E value of 1e^−10^ and minimum identity of >30%.

Probiotic genes were identified by homology-based analysis toward genes previously described as having probiotic features by Turpin et al. (19). Homology was predicted by BLASTP with a cutoff E value of 1e^−10^ and minimum identity of >35% and then manually curated. Antibiotic resistance genes (ARG) were computationally predicted within the BV-BRC platform (87) by a k-mer-based detection method and BLAST analysis and then manually curated. ARG were also predicted by the Antibiotic Resistance Genes Database (ARDB) tool, with an E value of 1 × 10^−10^ (88) and also retrieved by keywords search by using as query the term “antibiotic resistance” within the UniProtID entry list obtained by functional annotation. Each annotation was confirmed by interrogating InterPro (https://www.ebi.ac.uk/interpro/) to predict the functional analysis of the protein sequences.

Bacteriocins were predicted by uploading FASTA files of each isolate assembly into BAGEL4, a web service that uses a bacteriocin mining tool (89) to search gene clusters in prokaryotic DNA involved in the biosynthesis of *ri*bosomally synthesized and *p*osttranslationally modified *p*eptide*s* (RiPPs) and (unmodified) bacteriocins.

### Phenotypic characterization of *Weissella* strains.

**(i) Antimicrobial activity.** The six *Weissella* and *Periweissella* type strains in this study and Weissella viridescens ATCC 12706 were tested for inhibition of the potential gastrointestinal pathogens according to the method described by Verni and colleagues (90). Escherichia coli DSM 30083 (DSMZ, Germany), Bacillus megaterium F6 (91), and Listeria monocytogenes ATCC 19115 (ATCC, USA), belonging to the Culture Collection of the Department of Soil, Plants and Food Science, University of Bari, were used as tested strains. E. coli DSM 30083 and B. megaterium F6 were grown in Luria-Bertani (LB; Oxoid) medium for 24 h at 37 and 30°C, respectively, while L. monocytogenes was grown in brain heart infusion (BHI; Oxoid) for 24 h at 37°C. All *Weissella* strains were cultivated for 24 h at 30°C (final cell density of ca. 9 log CFU/mL). At the end of incubation, the cultures were centrifuged at 10,000 rpm for 10 min, and supernatants were recovered and neutralized at pH 6.2 using 2 N NaOH solution and sterile filtered with a 0.22-μm Millex-GP filter (Merck-Millipore, Darmstadt, Germany). An agar-well diffusion assay (92) was used to determine the antimicrobial activity of *Weissella* strain supernatants. The assay was carried out using the soft agar medium (5 mL), corresponding to the growth medium previously described, overlaid on 15 mL of agar-H_2_O (1.5%, wt/vol). Indicator strains were inoculated at 10^5^ CFU/mL, and 50 μL of cell-free supernatants were placed in wells of 5 mm in diameter. Fifty microliters of sterile MRS broth and chloramphenicol (final concentration, 0.1 g/L) were used as negative and positive controls, respectively.

**(ii) Screening for EPS-producing strains.** The six type strains of *Weissella* and *Periweissella* spp. and Leuconostoc pseudomesenteroides 20193^T^, used as a positive control, were grown on agar plates containing modified De Man, Rogosa, and Sharpe medium (MRS; Oxoid, Italy) supplemented with sterile sucrose (purity >99.5%; Sigma-Merck, Germany) solution to obtain a final concentration of 20 g/L. All strains were cultivated for 24 h as previously described and inoculated by spotting 10 μL of a ca. 10^8^ CFU/mL bacterial suspension on MRS-sugar agar medium. After incubation at 30°C for 48 h and 72 h, the strains which produced slimy colonies were recorded as capable of producing EPS.

**(iii) Survival of simulated gastrointestinal conditions.** Simulated gastric and intestinal fluids (SGF and SIF, respectively) were used as described by Fernández et al. (93) to evaluate the ability of the strains to resist the gastrointestinal tract. Stationary-phase-growth cells were harvested at 8,000 × *g* for 10 min, washed twice with physiologic solution, and suspended, at a cell density of ca. 9 log CFU/mL, in 50 mL of SGF which contained NaCl (125 mmol/L), KCl (7 mmol/L), NaHCO_3_ (45 mmol/L), and pepsin (3 g/L) (Sigma-Aldrich, St. Louis, MO, USA). The final pH was adjusted to 2.0, 3.0, and 8.0. The value of pH 8.0 was used to investigate the influence of the SGF components apart from the effect of low pH. The suspension was incubated at 37°C under anaerobic conditions and agitation with an orbital shaker to simulate peristalsis. Aliquots of the suspension were taken at 0, 90, and 180 min, and viable counts were determined. To mimic the effect related to the presence of the food matrix during gastric transit, cells were also suspended in reconstituted skimmed milk (RSM) (11% [wt/vol] solids) before inoculation of SGF at pH 2.0; after 180 min of gastric digestion, cells were harvested and suspended in SIF, which contained 0.1% (wt/vol) pancreatin and 0.15% (wt/vol) oxgall bile salt (Sigma-Aldrich Co.) at pH 8.0. The suspension was incubated as described above, and samples for total viable counts were taken at 0, 90, and 180 min.

**(iv) Adhesion assay.** The human colonic epithelial cell line Caco-2 purchased from IRCCS San Martino Polyclinic Hospital (Genoa, Italy) was used to assess the adhesion ability of studied strains. L. rhamnosus GG was included as a positive control for its capacity for binding to intestinal cells. A stock of Caco-2 cells was stored in liquid nitrogen and cultured for a few passages before performing the experiments. Caco-2 cells were cultured in Dulbecco’s modified essential medium, high glucose (DMEM; Euroclone S.p.A, Italy), supplemented with 10% inactivated fetal bovine serum, 1% l-glutamine, 1% antibiotic and antimycotic solution (Euroclone S.p.A, Italy), and 1% nonessential amino acid solution (Sigma-Aldrich, Italy) at 37°C under a humidified atmosphere containing 5% CO_2_. Cells were harvested twice a week up to 70 to 80% confluence using trypsin-EDTA solution. Cell density and viability were determined by a Scepter automated cell counter (Merck Millipore, Milan, Italy). The cells used for experimental protocols showed a mean viability of 90%. The adhesion ability assay was performed as described by Huang et al. (94) with slight modifications. Briefly, Caco-2 cells were seeded in 12-well cell culture plates (3.85 cm^2^) at a density of 5 × 10^4^ cells/cm^2^ (1.9 × 10^5^ cells/mL) and grown at 37°C with 5% CO_2_ for 7 days, changing the cell culture medium (1 mL/well) every 3 days to reach the complete confluence of cells. The relationship between optical density at 600 nm (OD_600_) (measured by using the Ultrospec 3100 spectrophotometer; Amersham Pharmacia Biotech, UK) and CFU per milliliter for each strain was determined as follows: bacterial cultures were grown overnight in MRS broth at 37°C, growth curve assays were performed by spreading serial dilutions of bacterial cultures at different OD_600_s (in the range of 0.1 to 1) on MRS agar plates and incubating them at 30°C, and then, the correspondence between OD_600_ and CFU per milliliter was recorded. For the adhesion assay, bacterial cultures grown overnight in MRS broth at 37°C were adjusted to the optical density corresponding to 10^8^ CFU/mL, as measured in the previously described growth curve assays (data not shown), to standardize the number of bacteria. Serial dilutions of the suspension were spread on an MRS agar plate to calculate the initial viable bacterial counts (CFU per milliliter). One milliliter of the same suspension was harvested by centrifugation at 5,000 rpm for 10 min and washed and resuspended in DMEM without supplements. Then, Caco-2 cell monolayers were washed twice with Dulbecco’s phosphate-buffered saline (PBS) without calcium and magnesium (Euroclone S.p.A, Italy), and 1 mL per well of bacterial suspension was added. After incubation for 2 h at 37°C in 5% CO_2_, unattached bacteria were removed by washing the monolayers three times with PBS. To lyse the cells, the monolayers were incubated for 15 min with 1 mL of 1% (vol/vol) Triton X-100 and serial dilutions of the resultant lysates were spread on an MRS agar plate to calculate the adhered bacterial counts (*A*). The percentage of adhesion was calculated according to the formula: % adhesion = [*A*(CFU/mL)/*I*(CFU/mL)] × 100.

**(v) Autoaggregation assay.** Autoaggregation was determined as described by Xu et al. (83). Bacterial cultures were grown overnight in MRS broth at 37°C and then harvested by centrifugation at 5,000 rpm for 10 min. Pellets were washed twice with PBS (pH 7.2) and resuspended in PBS solution to 1 × 10^8^ CFU/mL in the same solution. The initial absorbance was measured at 600 nm (OD_0_). A 1.5-mL amount of the solution was then transferred in 2-mL tubes and incubated at 30°C. Each hour, an aliquot (1 mL) from the top of the tubes was carefully removed, and its absorbance was read at 600 nm in a spectrophotometer (OD_tx_). The percentage of cellular aggregation was calculated according to the formula: % auto-aggragation = [(OD_0_ − OD_tx_)/OD_0_] × 100.

**(vi) Cell surface hydrophobicity.** Cell surface hydrophobicity was evaluated as described by Shangpliang et al. (95) with some modification. Bacteria were grown in MRS broth at 37°C for 24 h and then harvested by centrifugation at 5,000 rpm for 10 min. Pellets were washed twice with PBS solution (pH 7.2) and resuspended in PBS and adjusted to the optical density (OD_600_) corresponding to 10^8^ CFU/mL as previously determined (see “Adhesion assay” above) to standardize the number of bacteria (10^8^ CFU/mL) (*A*_0_). An equal volume of xylene was added to the suspension, and then the suspension was vortexed for 2 min. The 2-phase system was then incubated and left undisturbed for 1 h for phase separation. The organic phase was carefully removed, and the final absorbance of the aqueous phase was measured by determining the OD at 600 nm (*A*). The affinity for hydrocarbons (hydrophobicity) was reported as adhesion percent according to the formula [(*A*_0_ − *A*)/*A*_0_] × 100, where *A*_0_ and *A* are the absorbance before and after extraction with organic solvents, respectively.

**(vii) Hemolytic activity.** The hemolytic activity of the strains was tested by streaking bacterial cultures on Columbia agar plates (Oxoid, Altrincham, England) containing 5% (wt/vol) defibrinated sheep blood (Biolife, Milan, Italy) and anaerobically incubated at 30°C for 48 h (96). The presence of α- or β-hemolysis was indicated by the formation of greenish or clear zones around the colonies, respectively. Staphylococcus aureus DSM 799, Listeria monocytogenes DSM 20600, and L. rhamnosus GG were used as controls for β-, α-, and γ-hemolysis, respectively.

**(viii) Antibiotic resistance.** *Weissella* and *Periweissella* strains were grown in MRS broth for 24 h. The antibiotic resistance test was performed by agar-well diffusion assay and carried out on 15 mL of agar-H_2_O (1.5% [wt/vol]) overlaid with 5 mL of MRS agar with the inoculum of ca. 10^5^ CFU/mL for each strain. The bacterial susceptibility to antimicrobials was determined by a two-step analysis: since breakpoints have not been defined for *Weissella* and *Periweissella* genera, the susceptibility of the strains to antimicrobials was determined at the cutoff concentrations set for “other Gram-positive” bacteria by the FEEDAP panel (62): 1 mg/L ampicillin, 2 mg/L vancomycin, 4 mg/L gentamicin, 16 mg/L kanamycin, 8 mg/L streptomycin, 0.5 mg/L erythromycin, 0.25 mg/L clindamycin, 2 mg/L tetracycline, and 2 mg/L chloramphenicol. If no inhibition was shown after the first test, the MIC was assessed using concentrations 2.5-, 5-, and 10-fold higher than the cutoff values. The incubation time and temperature for both the steps were 24 h and 30°C, respectively. In this work, strains were considered resistant to a specific antimicrobial when the MIC was >10-fold the cutoff value described by the FEEDAP panel (62).

### Statistical analysis.

All experiments were performed in triplicate. The Shapiro-Wilk test to check the normality of the data and one-way analysis of variance (ANOVA) (*P *< 0.05) followed by Bonferroni’s *post hoc* test were performed in autoaggregation, adhesion, cell surface hydrophobicity, and gastrointestinal survival assays. The presence/absence of the response in all the triplicates was evaluated to express the results of the EPS production, antibiotic resistance, hemolysis, and antimicrobial activity assays.

### Data availability.

The whole-genome shotgun projects were deposited at DDBJ/ENA/GenBank under the following accession numbers (also listed in Table S2 in the supplemental material): *P. beninensis* LMG 25373^T^, JAGMVS010000000; *W. diestrammenae* DSM 27940^T^, JAGMVT010000000; *P. fabalis* LMG 26217^T^, JAGMVU010000000; *P. fabaria* LMG 24289^T^, JAGMVV010000000; *P. ghanensis* DSM 19935^T^, JAGMVW010000000; *W. uvarum* B18NM42^T^, JAGMVX010000000.
