# Supplementary material for: Probiotic Potential and Safety Assessment of Type Strains of Weissella and Periweissella Species
Source: Microbiol Spectr. 2023 Feb 27;11(2):e03047-22. doi: 10.1128/spectrum.03047-22 (PMC10100829; doi:10.1128/spectrum.03047-22)
Supplement: Supplemental file 1 — Tables S1 and S2. Download spectrum.03047-22-s0001.pdf, PDF file, 0.4 MB [file spectrum.03047-22-s0001.pdf]

Supplementary Table 1. Summary of bacteriocins in the genomes of *Weissella* and *Periweissella* strains as predicted by BAGEL4 and hit score obtained by homology search against BAGEL4 protein database

Strain	Area Of Interest	Class	Position	Protein ID	Hit score
***W. diestrammenae* DSM 27840^T**	AOI_1	114.2; Lacticin_Q	NODE_5:89606..109744	KAR27_07735	Evalue=1.36e^{-14} match=55.319%
***P. fabalis* LMG 26217^T**	AOI_1	43.2; Bovicin_255_peptide	NODE_13:1055..20400	KAR41_01340	Evalue = 4e^{-13} identity = 52.27 %
***W. uvarum* B18NM42^T**	AOI_1	66.2; Divercin_V41_(DvnV41)	NODE_1:28820..49003	KAR63_00175	Evalue=3.53e^{-12} match=40.00%
	AOI_2	153.2; MR10B	NODE_1:53868..73982	KAR63_00290	Evalue=3.36e^{-15} match = 53.488%
	AOI_3	31.1; Ericin_S	NODE_3:185729..210943	KAR63_05910	Evalue=1.11e^{-11} match=43.10%
	AOI_4	180.2; Plantaricin_S-alpha	NODE_13:8..10979	KAR63_03405 -03420-03425	Evalue = 3.66e^{-9} identity = 45.24 %

Supplementary Table 2. List of *Periweissella* and *Weissella* strains and Accession as deposited in DDBJ/ENA/GenBank used in this study

Species	Type strain	Reference Accession	Assigned by
P. beninensis	DSM 22752^T	JAGMVS010000000	[1]
W. diestrammenae	DSM 27940^T	JAGMVT010000000	[2]
P. fabalis	LMG 26217^T	JAGMVU010000000	[1]
P. fabaria	LMG 24286^T	JAGMVV010000000	[1]
P. ghanensis	DSM 19935^T	JAGMVW010000000	[1]
W. uvarum	B18NM42^T	JAGMVX010000000	[3]

References

1. Bello S, Rudra B, Gupta RS. 2022. Phylogenomic and comparative genomic analyses of *Leuconostocaceae* species: identification of molecular signatures specific for the genera *Leuconostoc*, *Fructobacillus* and *Oenococcus* and proposal for a novel genus *Periweissella* gen. nov. Int J Syst Evol Microbiol 72(3). https://doi.org/10.1099/ijsem.0.005284.
2. Oh SJ, Shin NR, Hyun DW, Kim PS, Kim JY, Kim MS, Yun JH, Bae JW. 2013. *Weissella diestrammenae* sp. nov., isolated from the gut of a camel cricket (*Diestrammena coreana*). Int J Syst Evol Microbiol. 63(8):2951-2956. https://doi.org/10.1099/ijs.0.047548-0.
3. Nisiotou A, Dourou D, Filippousi ME, Banilas G, Tassou C. 2014. *Weissella uvarum* sp. nov., isolated from wine grapes. Int J Syst Evol Microbiol. 64(11):3885-3890. https://doi.org/10.1099/ijs.0.066209-0.
